# 基于TCGA数据库的中央型与周围型肺鳞癌基因表达差异性研究

**DOI:** 10.3779/j.issn.1009-3419.2019.05.04

**Published:** 2019-05-20

**Authors:** 伟婷 李, 永文 李, 洪兵 张, 颖 李, 茵 袁, 颢 宫, 森 韦, 红雨 刘, 军 陈

**Affiliations:** 1 300052 天津，天津医科大学总医院肺部肿瘤外科 Department of Lung Cancer Surgery, Tianjin 300052, China; 2 天津市肺癌研究所，天津市肺癌转移与肿瘤微环境重点实验室 Tianjin Key Laboratory of Lung Cancer Metastasis and Tumor Microenvironment, Tianjin Lung Cancer Institute, Tianjin Medical University General Hospital, Tianjin 300052, China

**Keywords:** 肺肿瘤, 中央型, 周围型, 预后, Lung neoplasms, Central type, Peripheral type, Prognosis

## Abstract

**背景与目的:**

肺癌是一种具有高发病率与高死亡率的恶性肿瘤疾病，最常见的类型为非小细胞肺癌（non-small cell lung cancer, NSCLC），其中肺鳞癌作为NSCLC中的一个亚型，具有特殊的病理学类型及其特定的治疗方法，根据临床表型不同又可分为周围型和中央型。本研究基于中央型和周围型肺鳞癌的临床差异进一步探索其基因水平的差异和其潜在的价值。

**方法:**

从癌症基因组图谱（The Cancer Genome Atlas, TCGA）数据库收集肺鳞癌数据集，下载临床信息资料及基因表达谱资料。整理资料，分析临床数据及相对应的基因信息。

**结果:**

在临床特征分析中发现，中央型肺鳞癌较周围型肺鳞癌更容易发生淋巴结转移（46.2%, 67/145 *vs* 28.9%, 26/90; *P*=0.019），而在性别、年龄、肿瘤大小、有无远处转移、TNM分期、表皮生长因子受体（epidermal growth factor receptor, *EGFR*）突变等方面未见明显差异。在基因表达水平分析中发现，中央型与周围型肺鳞癌具有1, 031个差异表达基因，其中，周围型与中央型相比，629个基因表达水平上调，402个基因表达水平下调。进一步富集分析显示差异表达基因主要体现在6个信号通路中，其中，刺激神经组织的配体-受体相互作用（neuroactive ligand-receptor interaction）通路是差异表达基因主要富集通路，其他差异表达基因主要与脂类代谢和糖代谢有关。相互作用网络分析显示，在表达上调差异基因中，肝细胞核因子1同源体A（hepatocyte nuclear factor 1 homeobox A, HNF1A）和细胞色素P450家族里的A亚家族发现的第四种酶（cytochrome p450 family, Cytochrome P450 3A4, CYP3A4）影响较为广泛，在表达下调差异基因中，人血清白蛋白（Albumin, ALB）与载脂蛋白A1（Apolipoprotein, APOA1）位于该作用网络的关键位置。

**结论:**

中央型和周围型肺鳞癌患者不仅在淋巴结转移发生率上存在临床特征的差异，而且在基因表达水平亦有明显的不同。其中，HNF1A、CYP3A4、ALB、APOA1位于差异基因相互作用网络的关键位置，有可能参与调控二者的差异表型（phenotypic difference）。

## Abstract (en)

**Background and objective:**

Lung cancer is a malignant tumor disease with high morbidity and high mortality. The non-small cell lung cancer (NSCLC) is the most common type, among them, lung squamous cell carcinoma own special pathological type and specific treatment, is a subtype of non-small cell lung cancer and can be divided into peripheral type and central type according to clinical phenotype. This study explores the differences in gene levels and their potential values based on clinical differences between central and peripheral in lung squamous cell carcinoma.

**Methods:**

The lung squamous cell carcinoma dataset was collected from The Cancer Genome Atlas (TCGA) database, clinical information and the corresponding gene expression profiles were downloaded. Then we further sort and analyze all these data.

**Results:**

In clinical characteristics analysis, result showed that central lung squamous cell carcinoma was more likely to metastasis with lymph node than peripheral lung squamous cell carcinoma (46.2%, 67/145 vs 28.9%, 26/90; *P*=0.019), while there were no significant differences in gender, age, tumor size, distant metastasis, tumor node metastasis (TNM) stage, and *EGFR* mutation. Gene expression analysis showed 1, 031 differentially expressed genes between central and peripheral lung squamous cell carcinoma, of which 629 genes were up-regulated and 402 genes were down-regulated (peripheral *vs* central). Further enrichment analysis showed differentially expressed genes were mainly riched in 6 signaling pathways. Among them, the neuroactive ligand-receptor interaction pathway was the main enrichment pathway of differentially expressed genes, and other differential expressed genes were mainly involved in lipid metabolism and glucose metabolism. The analysis of interaction network showed that hepatocyte nuclear factor 1 homeobox A (HNF1A) and cytochrome p450 family, Cytochrome P450 3A4 (CYP3A4) own widely effect in up-regulated genes, while ALB and APOA1 at the key positions of the network in down-regulated genes were

**Conclusion:**

Central and peripheral lung squamous cell carcinoma showed clinical phenotype difference not only reflected in the incidence of lymph node metastasis, but also in gene expression profiles. Among them, HNF1A, CYP3A4, ALB, APOA1 at the key position of the differential gene interaction network and maybe as regulatory factors in the phenotypic difference.

恶性肿瘤已成为世界性的公共卫生问题，其中，肺癌的发病率呈现逐年上升的趋势，其发病率在男性恶性肿瘤患者中居第一位，女性恶性肿瘤患者中居第二位，而其死亡率不论男女均位居恶性肿瘤的第一位^[1]^。在我国，肺癌已成为最常见的恶性肿瘤，每年新发病例大约70多万，而死亡病例约60余万。肺癌从组织学上主要分为小细胞肺癌（small cell lung cancer, SCLC）（占15%）和非小细胞肺癌（non-small cell lung cancer, NSCLC）（占85%）。其中NSCLC又主要分为腺癌（adenocarcinoma)、鳞癌（squamous cell carcinoma, SCC)，和大细胞癌（large cell lung cancer, LCLC）。全世界每年死于肺鳞癌的患者高达40万人^[2]^。近年来，肺癌的治疗研究也在不断进步，例如表皮生长因子受体酪氨酸激酶抑制剂（epidermal growth factor receptor tyrosine kinase inhibitors, EGFR-TKIs），人间变性淋巴瘤激酶（anaplastic lymphoma kinase, ALK）等为代表的分子靶向药为治疗带来了新的进展^[3]^。患者的生存时间和生存质量得到了较大的提高。有研究^[4]^指出，肺鳞癌发病率低但侵袭性相对肺腺癌高，大部分肺鳞癌在诊断时已处于晚期，由于肺鳞癌在的敏感突变率低于肺腺癌，EGFR-TKI缓解率通常也只有30%，明显低于肺腺癌^[5]^，分子靶向治疗药物并不适用于大多数的肺鳞癌患者，所以晚期肺鳞癌患者的标准治疗仍然是细胞毒性化疗，肺鳞癌与肺腺癌具有某些相似的临床特征，相比之下，鳞癌患者在治疗上的进展有限^[6]^。

肺鳞癌是一类起源于支气管上皮、病理显示有角化和（或）细胞间桥存在的恶性上皮肿瘤，约占NSCLC的40%。肺鳞癌多发于吸烟者，有研究表明吸烟患肺癌的危险性是不吸烟者的4.97倍^[7]^。肺鳞癌多发生在段支气管及次段大支气管，故多为中央型^[8]^，中央型具有向管腔内生长、癌组织易变性、坏死形成空洞或发生出血的特点。近年来周围型肺鳞癌发病率在上升，约占肺鳞癌总数的50%。周围型肺鳞癌常见血管、胸膜侵犯，较少淋巴结转移，肺泡充盈型生长方式的周围型鳞癌预后较好。中央型和周围型肺鳞癌组织学上并无差别^[9]^。有文献^[10]^报道肺鳞癌中央型和周围型不仅在肿瘤大小和淋巴结转移上存在差异，其生物学特性如不同肿瘤的侵袭程度亦不同。因此，在分析周围型与中央型肺鳞癌患者临床特征的基础上，进一步分析其分子生物学特征的差异，必将为肺鳞癌患者的精准诊治奠定基础。本研究拟通过癌症基因组图谱（The Cancer Genome Atlas, TCGA）公共数据集，在分析研究中央型肺鳞癌及周围型肺鳞癌的临床特征差异的基础上，进一步分析其在基因分子水平的差异，为进一步明确中央型和周围型肺鳞癌在发生发展中的作用机制不同和可能的靶向治疗提供线索和思路。

## 1 材料与方法

### 1.1 数据资料收集

利用TCGA简易下载工具包从TCGA数据库（https://tcga-data.nci.nih.gov/tega/）下载肺鳞癌数据集的临床资料及RNASEqV2信息。

### 1.2 数据集筛选和临床参数及基因信息相关性研究

根据表达谱数据，对样本的中央型肺鳞癌及周围型肺鳞癌进行临床信息整理和对应基因信息的筛选，仅保留TCGA数据集中包含临床参数和对应的基因信息的病例。

### 1.3 基因差异分析

采用R软件语言的“cluster Profiler”，“pathview”，“pheatmap”，“vegan”，“volcano”的程序包辅助编程对RNA-sequence查找差异基因，并对差异基因（differential gene, DEFs）进行KEGG富集分析。GCBI网站（www.gcbi.com.cn）进行基因间相互作用分析。

### 1.4 统计学方法

使用SPSS 21.0软件进行统计学分析。临床信息相关性分析，组间比较采用*χ*^2^检验及*Fisher*确切概率法，采用乘积极限法（*Kaplan-Meier*）绘制生存曲线、对数秩检验（*Log-rank*）比较不同样本的生存曲线。*P* < 0.05为差异有统计学意义。

## 2 结果

### 2.1 中央型和周围型肺鳞癌患者的临床病理特征分析

本研究从TCGA数据集下载、整理、分析了240例肺鳞癌患者的临床数据和其对应的基因信息，其中，男性181例（75.4%, 181/240)，女性59例（24.6%, 59/240），平均年龄（66.528±8.828）岁；存在吸烟史的225例（95.3%），无吸烟史的11例（4.7%）；中央型147例（61.25%），周围型93例（38.75%）。如表 1所示，240例中央型和周围型肺鳞癌患者的临床理特征相关性分析结果显示，中央型和周围型肺鳞癌患者在性别、年龄、肿瘤大小、有无远处转移、TNM分期、*EGFR*突变方面均无统计学差异，而在淋巴结转移发生率上，中央型较周围型肺鳞癌患者更容易发生淋巴结转移（中央型：67/145，46.2%，周围型：26/90，28.9%）（*P*=0.019）。进一步采用*Kaplan-Meier* Plotter方法分析中央型和周围型肺鳞癌患者的生存差异，如图 1所示，二者在生存时间上未见明显的统计学不同（*Log-rank*检验，*P*=0.298，图 1）。

**1:**
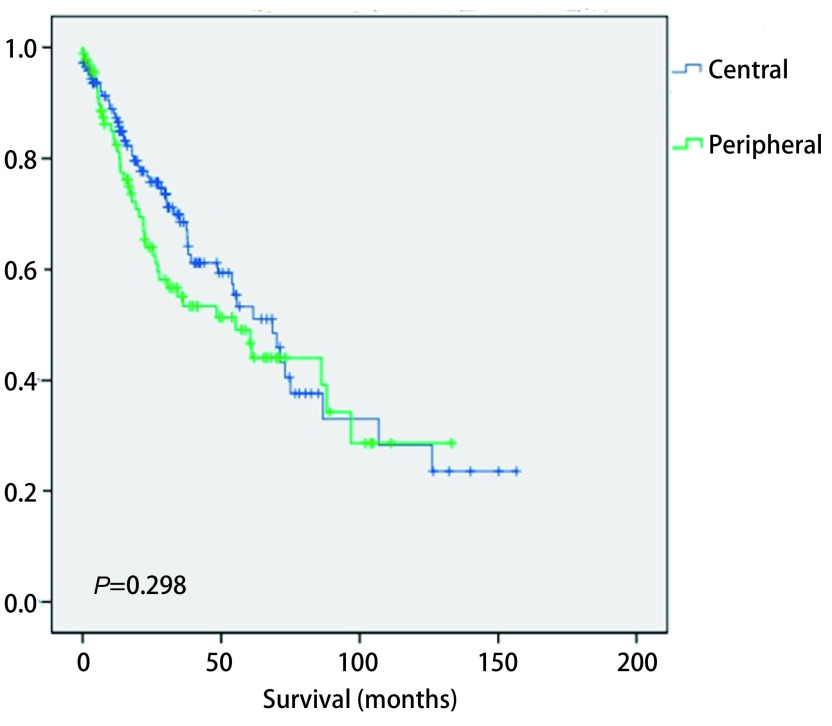
中央型和周围型肺鳞癌的生存曲线比较 Comparison of survival curves between central and peripheral squamous cell carcinoma

**1:** 中央型和周围型肺鳞癌患者的相关临床资料分析 Analysis of clinical data of patients with central and peripheral lung squamous cell carcinoma

	*n*	Peripheral	Central	*P*
Gender				
Female	59	25	34	0.511
Male	181	68	113	
Age (year)				
＜60	45	12	36	0.065
≥60	188	77	108	
Primary site				
Left	97	38	59	0.317
Right	125	51	74	
History other malignancy				
Yes	19	6	13	0.504
No	221	87	134	
T stage				
T1, T2	193	72	121	0.352
T3, T4	47	21	26	
N stage				
N0	142	64	78	0.019
N1，N2	93	26	67	
M stage				
M0	217	86	131	0.266
M1, M1b	3	2	1	
TNM stage				
StageⅠ-Ⅱ	192	75	117	0.38
Stage Ⅲ-Ⅳ	45	18	27	
EGFR mutation				
Yes	5	3	2	0.572
No	196	74	122	
Residual tumor				
Yes	19	5	14	0.413
No	211	85	126	
Tobacco				
Yes	225	90	135	0.193
No	11	3	8	
Some items in this table have missing values Age (-7), Primary site (-18), N stage (-5), M stage (-20), TNM stage (-3), EGFR mutation (-39), Residual tumor (-10), Tobaco (-4); TNM: tumor node metastasis; EGFR: epidermal growth factor receptor.				

### 2.2 中央型和周围型肺鳞癌患者的基因表达水平的分析

本研究运用R语言“pheatmap”、“vegan”、“volcano”的程序包在中央型和周围型肺鳞癌两组患者中筛选了差异表达基因，结果显示二者存在1, 031个差异表达基因，其中，周围型鳞癌相对于中央型鳞癌有629个基因表达水平上调，402个基因表达水平下调（图 2A-图 2B）。

**2:**
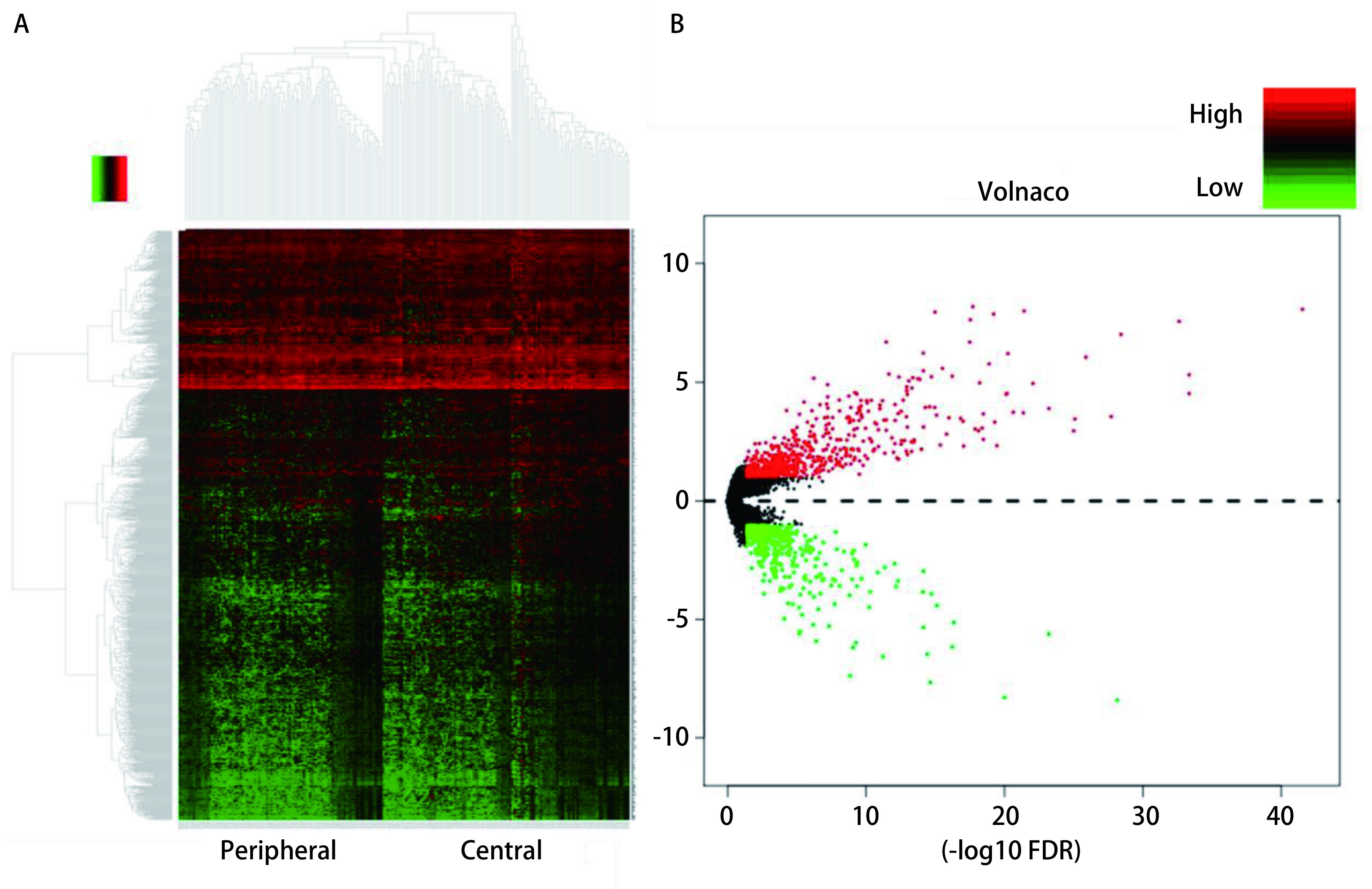
R程序分析出中央型和周围型肺鳞癌之间存在1, 031个有意义的差异基因，周围型鳞癌相对于中央型鳞癌有629个基因表达水平上调，402个基因表达水平下调。 The R program analyzed 1, 031 significant differential genes between central and peripheral lung squamous cell carcinoma. There are with 629 genes up-regulated and 402 genes were down-regulated (Peripheral vs central).

### 2.3 中央型和周围型肺鳞癌差异基因的相关KEGG富集信号通路分析

为了更进一步了解造成二者基因差异的相关生物进程，本研究采用R软件的“cluster Profiler”、“pathview”程序包对中央型和周围型肺鳞癌的1, 031个差异表达基因信息进行KEGG pathway富集分析，结果显示差异基因主要富集在6个信号通路中：刺激神经组织的配体-受体相互作用（neuroactive ligand-receptor interaction），青少年糖尿病的成年发病（maturity onset diabetes of the young），脂肪消化与吸收（fat digestion and absorption），细胞色素P450对异种生物的代谢作用（metabolism of xenobiotics by cytochrome P450），胆固醇代谢（cholesterol metabolism），唾液分泌（salivary secretion）（图 3）。其中，差异表达基因主要富集在刺激神经组织的配体−受体相互作用（neuroactive ligand-receptor interaction）信号通路上，而另外5个信号通路主要与代谢（糖、脂肪）有关。因此，KEGG pathway（Kyoto Encyclopedia of Genes and Genomes）富集的结果显示，周围型和中央型肺鳞癌的功能差异可能主要集中在神经分泌及代谢的差异上。

**3:**
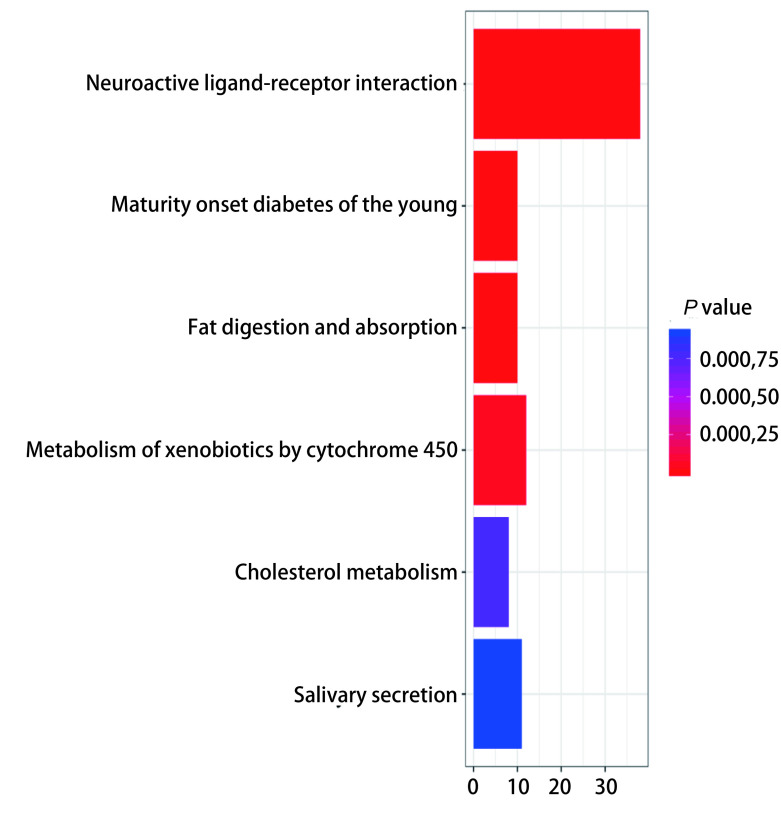
R程序针对周围型和中央型肺鳞癌的1, 031个差异基因分析其KEGG pathway富集（即信号通路富集） R program for KEGG enrichment (Signal pathway enrichment) of 1, 031 differential genes in peripheral and central lung squamous cell carcinoma

### 2.4 中央型及周围型肺鳞癌差异基因的相互作用网络分析

进一步将上述筛选出的629个表达水平上调基因和402个下调基因，录入GCBI网站（www.gcbi.com.cn），分析其所编码的蛋白之间的相互作用，绘制出了差异基因的相互作用网络图（图 4）。结果显示，即肝细胞核因子1同源体A（hepatocyte nuclear factor 1 homeobox A, HNF1A）和人血清白蛋白（Albumin, ALB）在其中影响较为广泛，位于网络中心的重要节点。KEGG pathway（Kyoto Encyclopedia of Genes and Genomes）分析已经显示差异基因主要富集在刺激神经组织的配体-受体相互作用通路上，而*HNF1A*和*ALB*基因也主要富集在此通路上，提示*HNF1A*和*ALB*基因可能通过激神经组织的配体-受体相互作用通路参与肺鳞癌中央型和周围型的差异调控。*HNF1A*基因是糖尿病发生的重要基因之一，并且参与脂类代谢。*ALB*基因编码的蛋白是人类血液中最丰富的蛋白质。这种蛋白质在调节血浆胶体渗透压方面起作用，并且作为包括激素、脂肪酸和代谢物以及外源药物的广泛内源分子的载体蛋白。差异基因相关作用网络中还显示胞色素P450（cytochrome P450）家族中A亚家族中的众多家族成员（如CYP3A4，CYP2B6；Cytochrome P450 2B6，CYP2A6 Cytochrome P450 2A6，CYP2A13；Cytochrome P450 2A13），以及载脂蛋白A1（apolipoprotein, *APOA1*）基因的多个同源体（APOA1、AP2、APOA3、APOA4等）也处于该相互作用网络的关键位置，与多个基因存在相互作用的关系。细胞色素P450（cytochromeP450或CYP450，简称CYP450）是一类主要存在于肝脏、肠道中的单加氧酶，主要参与内源性物质和包括药物、环境化合物在内的外源性物质的代谢。*APOA1*基因编码载脂蛋白A1，是血浆高密度脂蛋白（high density lipoprotein, HDL）的主要蛋白成分，是脂代谢中重要的参与者。由此可见，差异基因相关作用网络图进一步验证了，代谢相关信号通路在肺鳞癌周围型和中央型的功能差异中扮演着重要的角色。

**4:**
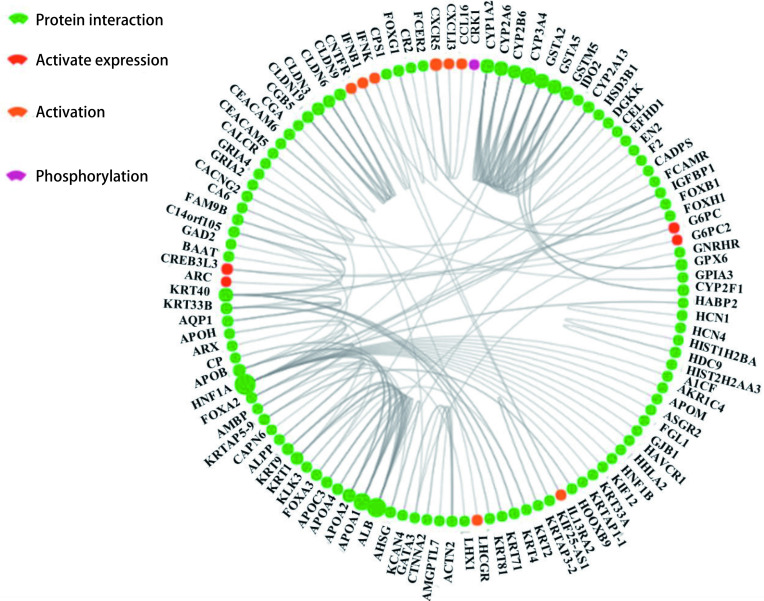
GCBI网站对分析了1, 031个差异基因编码的蛋白之间的相互作用 GCBI website analyzed the interaction between proteins encoded by 1, 031 differential genes

## 3 讨论

肺癌是我国发病率、死亡率最高的恶性肿瘤^[1]^。有研究^[1, 11]^显示，仅在2017年城市居民肺癌患者高达222, 500人，而因肺癌死亡的人也高达155, 870人。吸烟、遗传、环境污染、职业暴露、饮食等多种因素不同程度影响着肺癌的发生发展^[9]^。目前，治疗肺癌的方法有手术、化疗、放疗、分子靶向治疗、介入治疗和其他治疗（免疫治疗、中医药治疗、光动力学治疗、基因治疗、支持治疗）等^[12]^。而针对肺鳞癌患者主要采取手术治疗的方式^[13]^，而且中央型肺鳞癌的全肺切除率高于周围型肺鳞癌^[14]^。肺鳞癌是一类起源于支气管上皮、病理显示角化和（或）细胞间桥的恶性上皮瘤，多为中央型。虽然周围型肺鳞癌与中央型肺鳞癌在临床表现及病理组织学特点存在一定的差异，但其分子机制是否存在差异尚不明确。鳞癌与腺癌的分子生物学特性存在明显的差异，如有研究报道在肺鳞癌中，并未发现*KRAS*基因突变与*EGFR*基因突变有相关性^[15, 16]^，这点与腺癌有明显不同，不过并不排除在肺鳞癌中*KRAS*和*EGFR*突变较低的缘故。肺鳞癌的发生与吸烟有强相关性^[17]^，当然也离不开多种生物遗传事件的作用，本文主要探讨了中央型和周围型鳞癌的临床特征以及基因分子水平的差异。首先在中央型和周围型肺鳞癌患者的临床理特征相关性分析中我们发现中央型肺鳞癌较周围型更容易发生淋巴结的转移，这与文献报道的肺鳞癌中央型和周围型淋巴结转移上存在差异相一致，但在该研究中，与文献报道不同的是我们并没有发现二者在肿瘤大小上存在一定的差异^[10]^，但由于数据来源的不同，其原因是很难分析的。

在分子生物学特征的分析中，我们基因集富集分析（gene set enrichment analysis, GSEA）从全局水平分析不同表型的基因表达变化，发现了差异基因主要富集在6个信号通路中：刺激神经组织的配体−受体相互作用，青少年糖尿病的成年发病，脂肪消化与吸收），细胞色素P450对异种生物的代谢作用，胆固醇代谢和唾液分泌，从而揭示二者的差异主要在生物体发育和分化的机制上。目前的相关研究报道较少，一项全基因组关联研究显示15号染色体15q25.1是肺癌的主要易感区域，其中表达数量定位点（expression quantitative trait loci, eQTL）的功能分析结果显示，刺激神经组织的配体−受体相互作用（neuroactive ligand-receptor interaction）与肺癌发表风险有关，可能作为主要通路参与了肺癌的发生^[18]^。此外，亦有文献报道脂肪组织和脂肪细胞分泌的信号分子和代谢物，尤其是在肥胖状态下，直接或间接刺激抗凋亡作用被认为是癌症进展的重要因素^[19, 20]^。在糖的代谢方面，文献报道与大多数体细胞不同，癌细胞消耗大量的葡萄糖，依靠有氧糖酵解产生ATP，Otto Warburg对这一现象进行了描述，称之为“瓦氏效应”（Warburg effect）^[21, 22]^。因此，我们有理由相信上文中提到的差异基因富集信号通路如青少年糖尿病的成年发病（maturity onset diabetes of the young）、脂肪消化与吸收（fat digestion and absorption）、细胞色素P450对异种生物的代谢作用（metabolism of xenobiotics by cytochrome P450）、胆固醇代谢（cholesterol metabolism）、唾液分泌（salivary secretion）参与了周围型和中央型肺鳞癌表型差异的形成。

进一步相互作用网络分析显示HNF1A、CYP3A4、ALB与APOA1位于该该差异基因作用网络的关键位置。而*HNF1A*和*ALB*基因也主要富集在刺激神经组织的配体-受体相互作用通路上，提示*HNF1A*和*ALB*（Albumin，人血清白蛋白）基因可能通过激神经组织的配体-受体相互作用通路参与肺鳞癌中央型和周围型的差异调控；CYP3A4与APOA1分别主要参与内源性物质和包括药物、环境化合物在内的外源性物质的代谢和脂代谢，这进一步证实了KEGG富集信号通路分析的结果。HNF1A是编码肝细胞核因子1同源体A，这是一种在人类肝脏、胰腺、肾脏和肠道中表达的转录因子^[23]^。HNF1A是发育成熟胰腺中调控转录因子通路的重要成员^[24]^，并且HNF1A参与糖尿病和一些肝病的发生。*ALB*基因主要编码人血清白蛋白^[25]^，是人类血液中最丰富的蛋白质。人血清白蛋白在调节血浆胶体渗透压方面起主要作用，ALB主要结合与水，阳离子（如Ca^2+^、Nat和Kt），脂肪酸，激素，胆红素，甲状腺素（T4）和药物（包括巴比妥酸盐）结合，是包括激素、脂肪酸和代谢物以及外源药物的广泛内源分子的载体蛋白。文献报道在NSCLC中，血清中CRP/Alb可以作为肺癌患者化疗总生存期的一个预后因素^[26]^，而NSCLC术前白蛋白球与蛋白评分可以作为其预后的一个重要因素^[27]^，而且C反应蛋白与白蛋白（C-reactive protein/Albumin, CRP/ALB）的比值有可能是肺癌独立于病理分型和临床分期的一个预后指标^[28]^。

细胞色素P450（cytochrome p450 family, CYP450）是位于线粒体膜或内质网上的一组混合功能氧化酶系统的末端氧化酶。它们在内源性和外源性分子的代谢中起着至关重要的作用^[29]^。CYP450酶是所有器官中最重要的代谢酶家族。除了在大多数内源性化合物和外来生物的失活中起作用外，它们还介导大多数致癌物质氧化及代谢^[30]^，也因为它在细胞中参与多条代谢作用，也有人考虑结合此特性来参与心血管疾病或是癌症的治疗^[31]^。*APOA1*基因位于11号染色体11q23-q24，其编码的蛋白质参与多种生物功能，可被分离成一种稳定因子（Prostaglandin I2, PGI2）；HDL由APOA1编码，参与机体多种代谢功能，已有研究表明APOA1在肺癌患者中呈现较低水平表达^[32]^。在NSCLC接受顺铂化疗的患者中，APOA1的水平可以作为预测患者中生存期的预后因素^[33]^。在诊断方面，APOA1也有可能作为一个生物标志物用于临床诊断^[34]^。此外，在乳腺癌中，APOA1会增加乳腺癌的风险，它的多态性（APOA1-75G/A和+83C/T）与乳腺癌的发病相关^[35]^。

总之，本研究采用现有的TCGA数据库，对中央型和周围型肺鳞癌的差异进行了初步研究，发现二者不仅在淋巴结转移发生率上存在临床特征的差异，而且在基因表达水平亦有明显的不同。其中，HNF1A、CYP3A4、ALB、APOA1位于差异基因相互作用网络的关键位置，有可能参与调控二者的差异表型（phenotypic difference），为进一步探讨中央型和周围型肺鳞癌的发病机制和个体化治疗提供新的思路。

## References

[1] Siegel RL, Miller KD, Jemal A (2017). Cancer Statistics, 2017. CA Cancer J Clin.

[2] Allemani C, Weir HK, Carreira H (2015). Global surveillance of cancer survival 1995-2009: analysis of individual data for 25, 676, 887 patients from 279 population-based registries in 67 countries(CONCORD-2). Lancet.

[3] Ai X, Guo X, Wang J (2018). The rapies for advanced non-small cell lung cancer. Oncotarget.

[4] Shundo Y, Takahashi T, Itaya A (2011). Clinical study of forty-two patients who underwent resection for pulmonary adenosquamous carcinoma. Kyobu Geka.

[5] Shukuya T, Takahashi T, Kaira R (2011). Efficacy of gefitinib for non-adenocarcinoma non-small-cell lung cancer patients harboring epidermal growth factor receptor mutations: a pooled analysis of published reports. Cancer Sci.

[6] Iijima Y, Seike M, Noro R (2015). Prognostic significance of PIK3CA and SOX2 in Asian patients with lung squamous cell carcinoma. Int J Oncol.

[7] Liu ZQ, He F, Cai L (2015). A case-control study on smoking, passive smoking and the risk of lung cancer. Zhonghua Ji Bing Kong Zhi Za Zhi.

[8] Wang NJ, Sanborn Z, Arnett KL (2011). Loss-of-function mutations in Notch receptors in cutaneous and lung squamous cell carcinoma. Proc Natl Acad Sci U S A.

[9] Yao XJ, Liu LX (2014). Epidemiology and treatment of lung cancer. Xian Dai Zhong Liu Yi Xue.

[10] Li J, Li Y, Yu HB (2009). The relationship between the depth of invasion of central lung cancer and lymph node metastasis. Zhongguo Fei Ai Za Zhi.

[11] Chen W, Zheng R, Zhang S (2017). Cancer incidence and mortality in China in 2013: an analysis based on urbanization level. Chin J Cancer Res.

[12] Weeden CE, Solomon B, Asselin-Labat ML (2015). FGFR1 inhibition in lung squamous cell carcinoma: questions and controversies. Cell Death Discov.

[13] Lemjabbar-Alaoui H, Hassan OU, Yang YW (2015). Lung cancer: Biology and treatment options. Biochimica Et Biophysica Acta.

[14] Li XK, Liu ZY, Jiang YF (2017). Clinicopathological factors and prognosis between primary central and peripheral lung. Shandong Da Xue Xue Bao (Yi Xue Ban).

[15] Ramalingam SS, Blackhall F, Krzakowski M (2012). Randomized phase Ⅱ study of dacomitinib (PF-00299804), an irreversible pan-human epidermal growth factor receptor inhibitor, versus erlotinib in patients with advanced non-small-cell lung cancer. J Clin Oncol.

[16] Mahoney CL, Choudhury B, Davies H (2009). *LKB1*/*KRAS* mutant lung cancers constitute a genetic subset of NSCLC with increased sensitivity to MAPK and mTOR signalling inhibition. Br J Cancer.

[17] Islami F, Torre LA, Jemal A (2015). Global trends of lung cancer mortality and smoking prevalence. Translat Lung Cancer Res.

[18] Ji X, Bosse Y, Landi MT (2018). Identification of susceptibility pathways for the role of chromosome 15q25.1 in modifying lung cancer risk. Nat Commun.

[19] Nieman KM, Romero IL, Van Houten B (2013). Adipose tissue and adipocytes support tumorigenesis and metastasis. Biochimica Et Biophysica Acta.

[20] Park J, Morley TS, Kim M (2014). Obesity and cancer-mechanisms underlying tumour progression and recurrence. Nat Rev Endocrinol.

[21] Lathrop AE, Loeb L (1916). Further investigations on the origin of tumors in mice. Ⅲ. On the part played by internal secretion in the spontaneous development of tumors. J Cancer Res.

[22] Warburg O (1956). On the origin of cancer cells. Science.

[23] Harries LW, Ellard S, Stride A (2006). Isomers of the *TCF1* gene encoding hepatocyte nuclear factor-1 alpha show differential expression in the pancreas and define the relationship between mutation position and clinical phenotype in monogenic diabetes. Hum Mol Genet.

[24] Boj SF, Parrizas M, Maestro MA (2001). A transcription factor regulatory circuit in differentiated pancreatic cells. Proc Natl Acad Sci U S A.

[25] Lee P, Wu X (2015). Review. Modifications of human serum albumin and their binding effect. Curr Pharmaceut Design.

[26] Tomita M, Ayabe T, Maeda R (2017). Combination of advanced lung cancer inflammation index and c-reactive protein is a prognostic factor in patients with operable non-small cell lung cancer. World J Oncol.

[27] Li X, Qin S, Sun X (2018). Prognostic significance of albumin-globulin score in patients with operable non-small-cell lung cancer. Ann Surg Oncol.

[28] Chen C, Ge P, Bai YP (2017). CRP/Alb ratio in the prognosis of lung cancer. Jian Yan Yi Xue.

[29] Korobkova EA (2015). Effect of Natural Polyphenols on CYP Metabolism: Implications for Diseases. Chem Res Toxicol.

[30] He X, Feng S (2015). Role of Metabolic enzymes P450 (CYP) on activating procarcinogen and their polymorphisms on the risk of cancers. Curr Drug Metab.

[31] Chen C, Wang DW (2013). CYP epoxygenase derived EETs: from cardiovascular protection to human cancer therapy. Curr Top Med Chem.

[32] Hsu MC, Lee KT, Hsiao WC (2013). The dyslipidemia-associated SNP on the *APOA1*/*C3*/*A5* gene cluster predicts post-surgery poor outcome in Taiwanese breast cancer patients: a 10-year follow-up study. BMC Cancer.

[33] Zhao XS (2015). Predictive value of ApoA1 and α1-AT in the sensitivity of concurrent chemoradiotherapy for stage Ⅲ non-small cell lung cancer. Ji Ceng Yi Xue Lun Tan.

[34] Zhu Y, Wang X, Wang W (2018). Expression and influence of pentraxin-3, HbAlc and ApoA1/ApoB in serum of patients with acute myocardial infarction combined with diabetes mellitus type 2. Exp Ther Med.

[35] Liu XY, Cao WH, Wang Y (2011). Relationship investigation between *ApoA1* gene polymorphisms and breast cancer. Zhonghua Zhong Liu Fang Zhi Za Zhi.

